# Analysis of fixation materials in micro-CT: It doesn’t always have to be styrofoam

**DOI:** 10.1371/journal.pone.0286039

**Published:** 2023-06-14

**Authors:** Jan Scherberich, Anton G. Windfelder, Gabriele A. Krombach

**Affiliations:** 1 Department of Diagnostic and Interventional Radiology (Experimental Radiology), University Hospital Giessen, Giessen, Hesse, Germany; 2 Branch for Bioresources, Fraunhofer Institute for Molecular Biology and Applied Ecology IME, Giessen, Hesse, Germany; University of Florida, UNITED STATES

## Abstract

Good fixation of filigree specimens for micro-CT examinations is often a challenge. Movement artefacts, over-radiation or even crushing of the specimen can easily occur. Since different specimens have different requirements, we scanned, analysed and compared 19 possible fixation materials under the same conditions in the micro-CT. We focused on radiodensity, porosity and reversibility of these fixation materials. Furthermore, we have made sure that all materials are cheap and easily available. The scans were performed with a SkyScan 1173 micro-CT. All dry fixation materials tested were punched into 5 mm diameter cylinders and clamped into 0.2 ml reaction vessels. A voxel size of 5.33 μm was achieved in a 180° scan in 0.3° steps. Ideally, fixation materials should not be visible in the reconstructed image, i.e., barely binarised. Besides common micro-CT fixation materials such as styrofoam (-935 Hounsfield Units) or Basotect foam (-943 Hounsfield Units), polyethylene air cushions (-944 Hounsfield Units), Micropor foam (-926 Hounsfield Units) and polyurethane foam, (-960 Hounsfield Units to -470 Hounsfield Units) have proved to be attractive alternatives. Furthermore, more radiopaque materials such as paraffin wax granulate (-640 Hounsfield Units) and epoxy resin (-190 Hounsfield Units) are also suitable as fixation materials. These materials often can be removed in the reconstructed image by segmentation. Sample fixations in the studies of recent years are almost all limited to fixation in Parafilm, Styrofoam, or Basotect foam if the fixation type is mentioned at all. However, these are not always useful, as styrofoam, for example, dissolves in some common media such as methylsalicylate. We show that micro-CT laboratories should be equipped with various fixation materials to achieve high-level image quality.

## Introduction

Micro-computed tomography has been used as an imaging technique for many years [1] and has become a standard procedure for evaluating small samples in many disciplines. These include material sciences [2–8], mineral and soil science [9–12], or life sciences [13–15] like the analysis of small structures of various animal models [16–21]. Furthermore there are many more clinical use cases such as forensics [22] and bone analysis [23–28].

**Fig 1:**
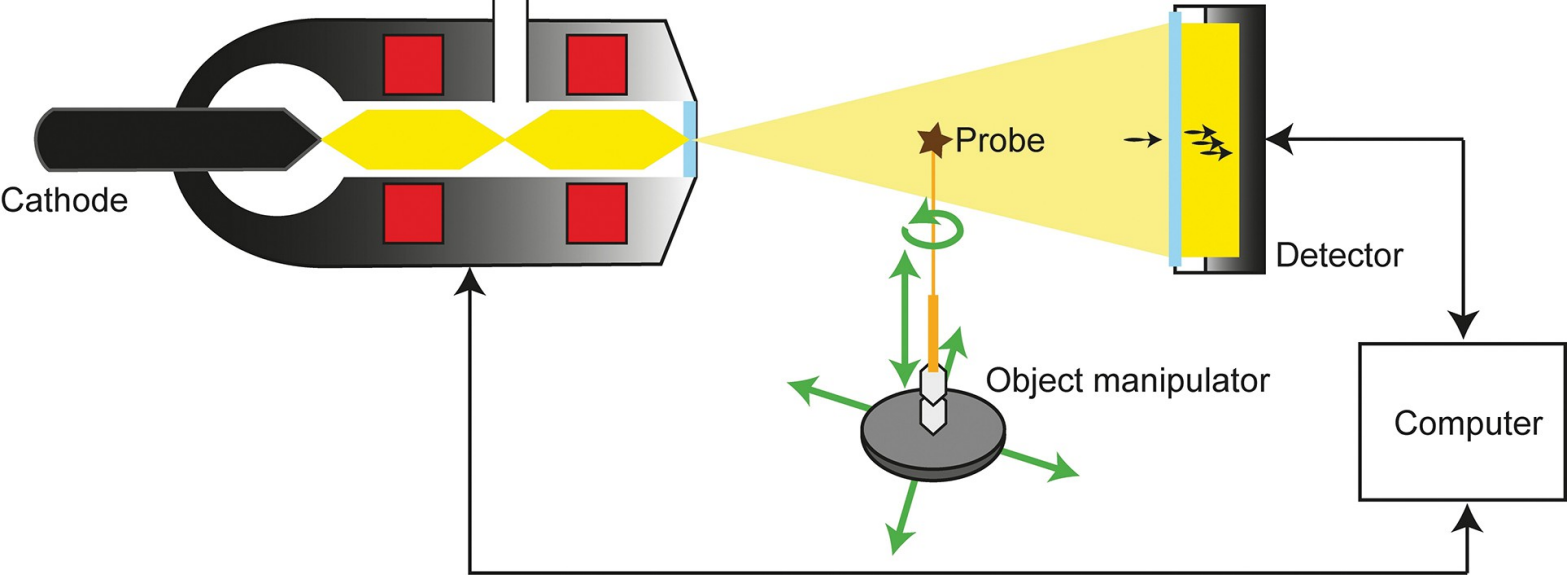
General setup for micro-CT measurements. An electron beam is emitted by the cathode (not true to scale). The probe is rotated after every scan in between the X-ray tube and a detector usually coupled with a camera. Magnification can be achieved by shifting the probe towards the X-ray beam.

X-rays are generated that pass through the sample and create a shadow image on a screen. By attenuating the X-ray radiation through the sample material and continuously changing the viewing angle, a three-dimensional construct of the sample can be formed that corresponds to the X-ray density of the material (Fig 1). Current micro-CT scanners achieve a resolution with a voxel size of only a few micrometres, down to the nanometre range. A voxel represents a three-dimensional pixel with an additional depth axis.

Depending on the device and settings, micro-CT measurements can take several hours. If the sample material shifts during this time, for instance due to rotation of the sample holder, the image will be blurred [29]. Therefore, a stable fixation of the sample is essential. However, the fixation material should not destroy the sample or make the later reconstruction more difficult. There are therefore several requirements for fixation materials and methods. They should either be barely visible under X-rays or produce such a homogeneous image that post-processing is not unnecessarily complicated. Ideally, the fixation should be reversible and not damage the sample. Foams and Styrofoam have proved particularly suitable for this purpose [30–32]. Often, dry samples are also fixed directly to a sample holder with superglue or similar adhesives [20, 33].

Especially soft or very filigree structures often pose a challenge during embedding. On the one hand, these samples need support in order not to collapse and on the other hand, materials that are too radiopaque unnecessarily complicate the later reconstruction.

Samples in micro-CT can be scanned surrounded by air or in a medium [34]. Solutions with samples can consist of water, saline, ethanol, or buffer solutions among other things. However, in many cases, prior dehydration by, for example, an ascending alcohol series, or by means of critical freeze-drying, may be useful. It has proven especially useful with samples in liquid to scan them in tubes that are as thin and fitting as possible and to fill gaps between the sample and the tube walls using further fixation materials as required.

**Fig 2:**
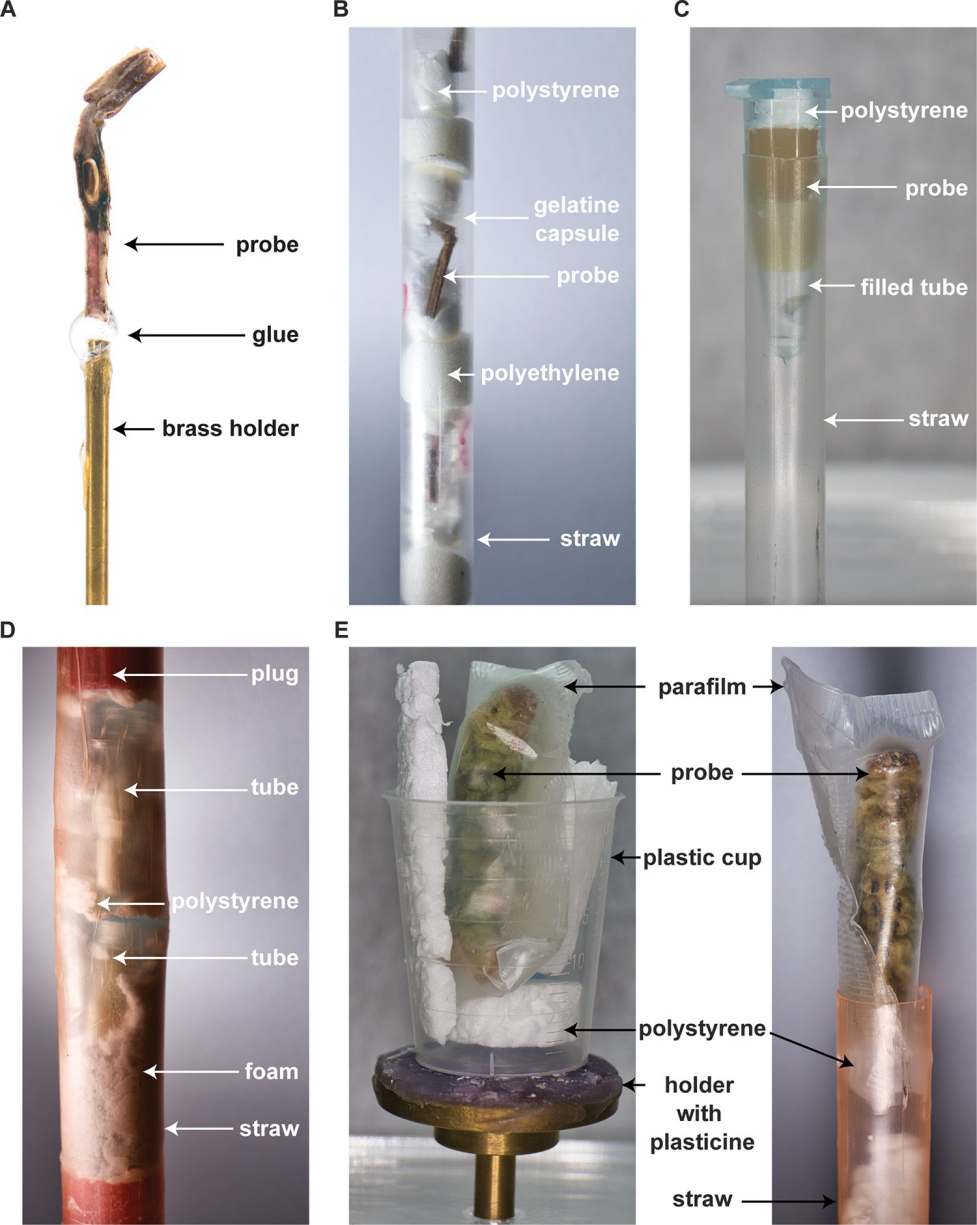
Fixation examples of micro-CT probes. (A) A bushcricket leg is fixed with instant glue to a brass holder. This fixation requires only a minimum of space and allows the specimen to be moved close to the X-ray source. (B) Example of multisample scanning of dry samples with gel capsules and polystyrene as spacer. (C) Moist samples can be fixed in a reaction tube with polystyrene. (D) Multisample scanning with reaction tubes inside a straw. Foam helps to prevent probe shifting. Plugging is useful to prevent expansion of the foam. (E) Parafilm can be used to prevent large samples from drying out. Open ends in the parafilm can simply be closed with ribbed forceps.

Usually, dry samples surrounded by air give the clearest and most contrasting image. It is therefore advisable, if possible, to glue them to a holder (Fig 2A) or to fix them with different materials, especially if they can be clamped in straws [35], gel capsules, or reaction tubes (Fig 2). For many specimens that are stored in solution, the contrast can be greatly improved if they are also temporarily removed from the solution and dabbed. If moist samples can be taken from the medium, parafilm fixation [30, 33, 36] is particularly suitable to prevent drying out during the scan duration (Fig 2E). In this case, it has proven useful to seal the parafilm with wide tweezers (Fig 2E). The sample wrapped in parafilm can then be fixed with other materials. Alternatively, it is also possible to seal the sample holder and leave a liquid reservoir inside [24]. For many samples, however, a scan in liquid is preferred to a scan in the air [19, 26, 33, 37]. Samples that need to remain in solution can be clamped and fixed in reaction vessels or plastic tubes, for example (Fig 2C). However, this is often not easy with fragile test materials (dry or wet) and requires other materials for support that do not interfere with the subsequent analysis if possible.

It is suspected that clear or particularly porous materials allow the best transmission of X-rays. Therefore, substances such as epoxy resin seem to be particularly suitable for sample fixation, but at the cost of reversibility of fixation. Styrofoam is also a popular fixation material, but it dissolves in substances such as methyl salicylate. In preliminary tests, PU samples (polyurethane; manufactured for standard MRI earplugs) have also been shown to be particularly radiolucent. It is therefore assumed that these are also particularly well suited for the fixation of micro-CT. Consequently, compromises with other fixation options must be considered depending on the requirements of the sample. The aim of this work is to provide an overview of suitable fixation options for different sample types.

## Material & methods

### Fixation and probe materials

In preliminary tests, PU samples and porous materials were found to be particularly permeable to X-rays. Therefore, three categories of materials were focused on in this work: PU from different manufacturers, as well as other porous and solid materials. To analyse the different materials, they were cut into 5 mm thick sample pieces of about 1 cm length with a core puncher and then fixed in a 0.2 ml reaction vessel without additional liquid. Liquid materials were filled directly into the reaction vessels. In order to calibrate the Hounsfield units as a measure of X-ray density, water phantoms were also scanned regularly (at least once a week).

### Scanning

Six samples were scanned from each material using a micro-CT system (SkyScan 1173, Bruker, Kontich, Belgium). The system contains a 40–130 kV X-ray tube and is equipped with a flat-panel sensor. The sensor consists of a CsI scintillator in which X-rays are converted to fluorescence and transmitted to a photodiode array. The electrical charge of the photodiodes accumulates for each pixel and thus reflects the light intensity. The spot size is <5 μm. The maximum spatial resolution is 7 μm (10% MTF). Projection images were captured per sample on a 16-bit 2240 × 2240 detector. The scans were all performed with the same settings at 45 kV, 150 μA and an exposure time of 1150 ms. By fixing several reaction tubes on top of each other in a thin plastic straw (shown in Fig 2D), a voxel size of 5.33 μm could be achieved. The samples were scanned with a 180° scan in 0.3° steps and the image noise was reduced by a fourfold averaging.

### Reconstruction and analysis

The reconstruction from projection images into 8 bit cross-sectional images was done with the software NRecon (NRecon 1.7.5.0, Bruker) with a smoothing parameter of 1, beam hardening 28% and a ring artefact correction of 5. A dynamic range of 0 to 0.03 was chosen for all reconstructions. If necessary, XY shifts were corrected using reference scans. CT Analyzer (CTan 1.20.8, Bruker) was used for binarization and analysis of the image series. Virtual sample cubes (2x2x2 mm) were taken, binarized (grey value threshold: starting from 10% of the dynamic range) and used for further analysis (X-ray density and porosity). Structure separation is calculated like the local thickness model [38] but for spaces instead of solid materials. In this algorithm spheres are drawn around points that are enclosed by the sphere and the sphere must be entirely bounded within the space.

### Statistical analysis

Six samples of each material were grouped and compared with other materials. The Brown-Forsythe and Welch ANOVA Test module with Dunnett’s T3 comparison test was used for statistical evaluation in the software Prism 9 (Prism 9.4.0, GraphPad Software, San Diego, USA).

## Results

In this study, different possible fixation materials for micro-CT samples were scanned and analysed. The sample fixation materials presented can be divided into three groups: solid and as homogeneous as possible materials, porous materials and in addition polyurethane foams as these were very promising in preliminary tests (S1 and S2 Figs). All three groups show significant differences in their radiolucency (p < 0.001). (Note that additional single scan samples are available in the supplemental data.)

### Polyurethane (PU)

**Fig 3:**
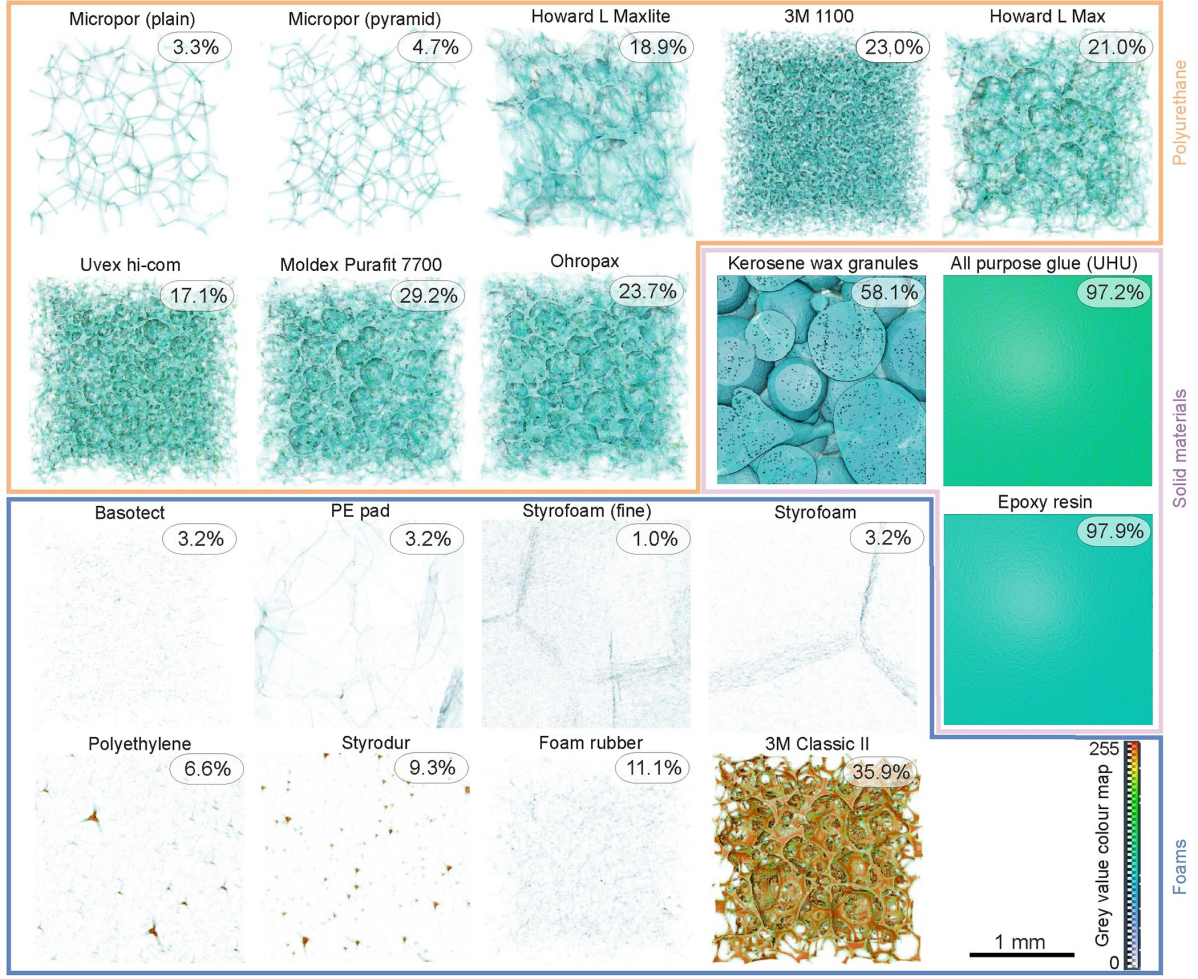
Rendered image of one exemplary cube of each sample. Grey values from the dynamic range of 0 to 0.03 are coded as colour values (0 to 255). The average binarised fraction of the sample is shown as a percentage for each material (n = 6 per material). Low binarised values are less visible in the evaluation than high values. Solid materials (outlined in purple) are the most radiopaque of the materials tested. Polyurethane samples (outlined in orange) are generally suitable as fixation materials but may often contain radiopaque particles. Other foams (outlined in blue) are particularly suitable as fixation material in the μCT. Basotect, the PE pad and Styrofoam should be particularly emphasised here. Scale: 1 mm.

**Fig 4:**
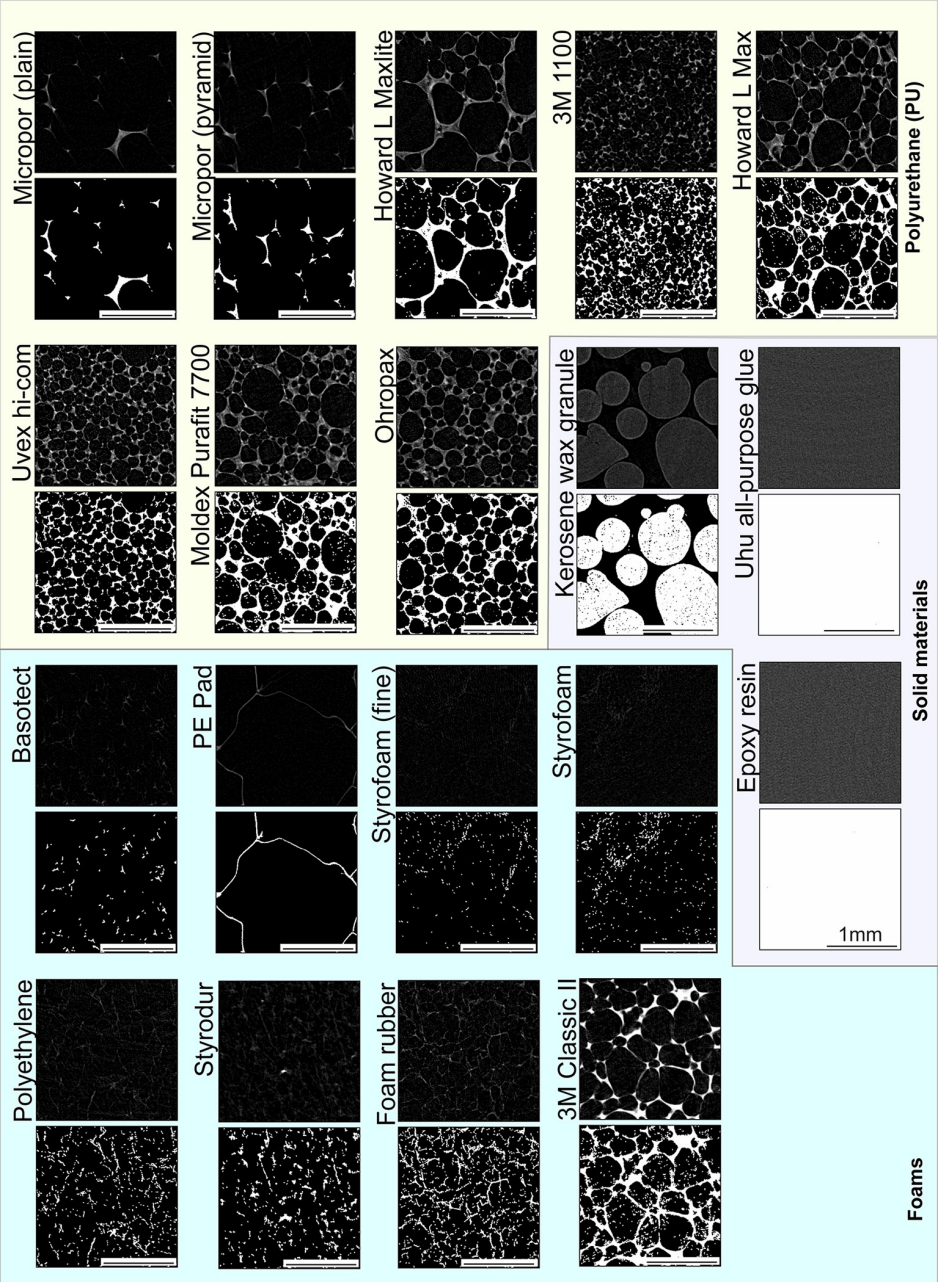
Reconstructed 2D cubes of the sample materials. The upper view shows an exemplary section of a sample type with a dynamic range of 0 to 0.03. The lower illustration shows the binarisation (starting from 10% of the dynamic range) of the same image section. In order to display a fixed micro-CT sample as easily as possible, the fixation material should not be visible (foams and polyurethane) or be easy to remove in post-processing (solid materials). Scale: 1 mm.

**Fig 5:**
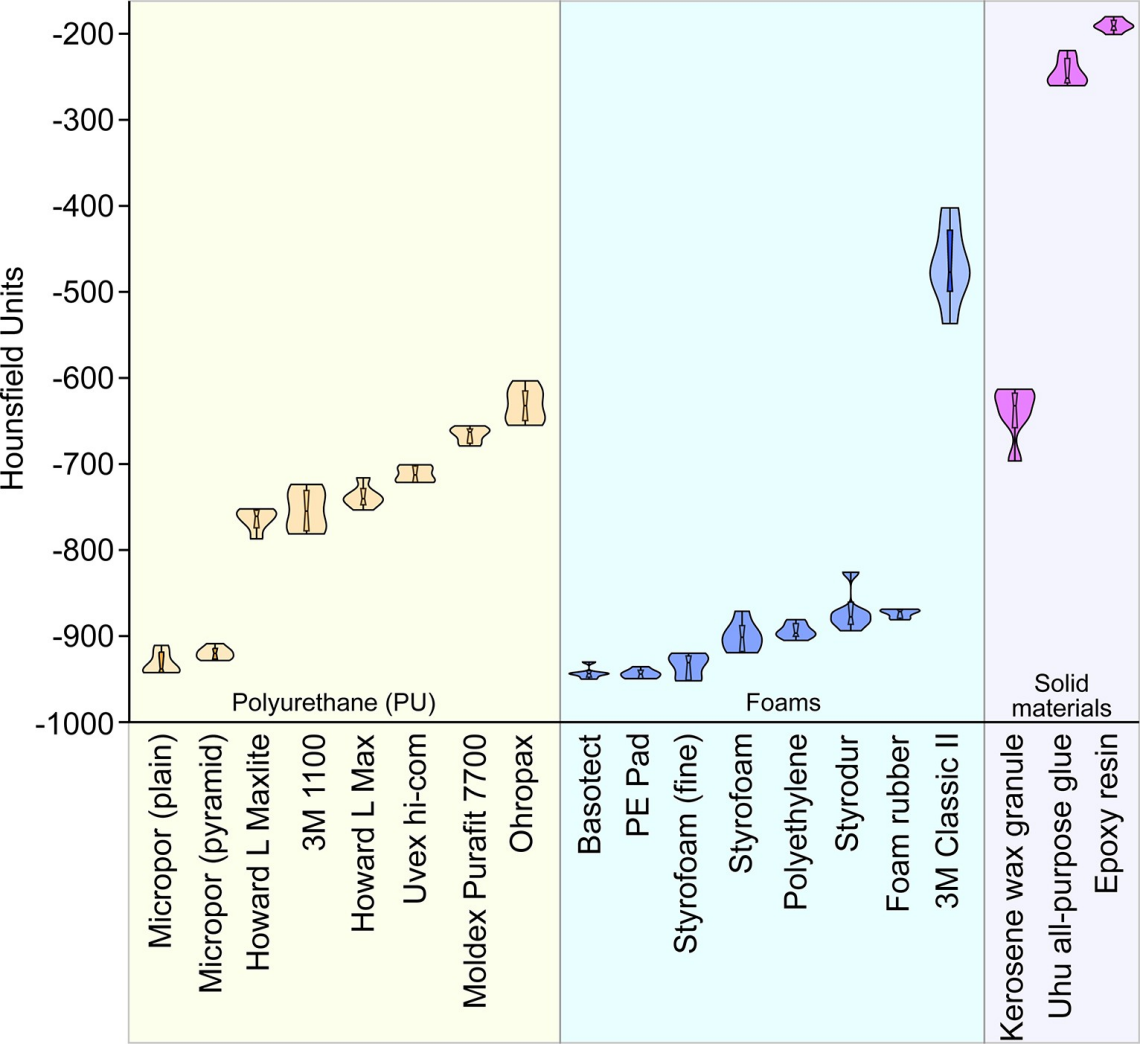
X-ray density in Hounsfield units of the fixation materials examined. Hounsfield Units (HU) is a measure of the X-ray density of a material, where -1000 HU corresponds to the density of air and 0 HU to the density of water. Foams (blue) usually have the lowest HU (radiolucent). Polyurethane (orange) and solid materials (purple) have very different radiopacities (n = 6 per material).

**Fig 6:**
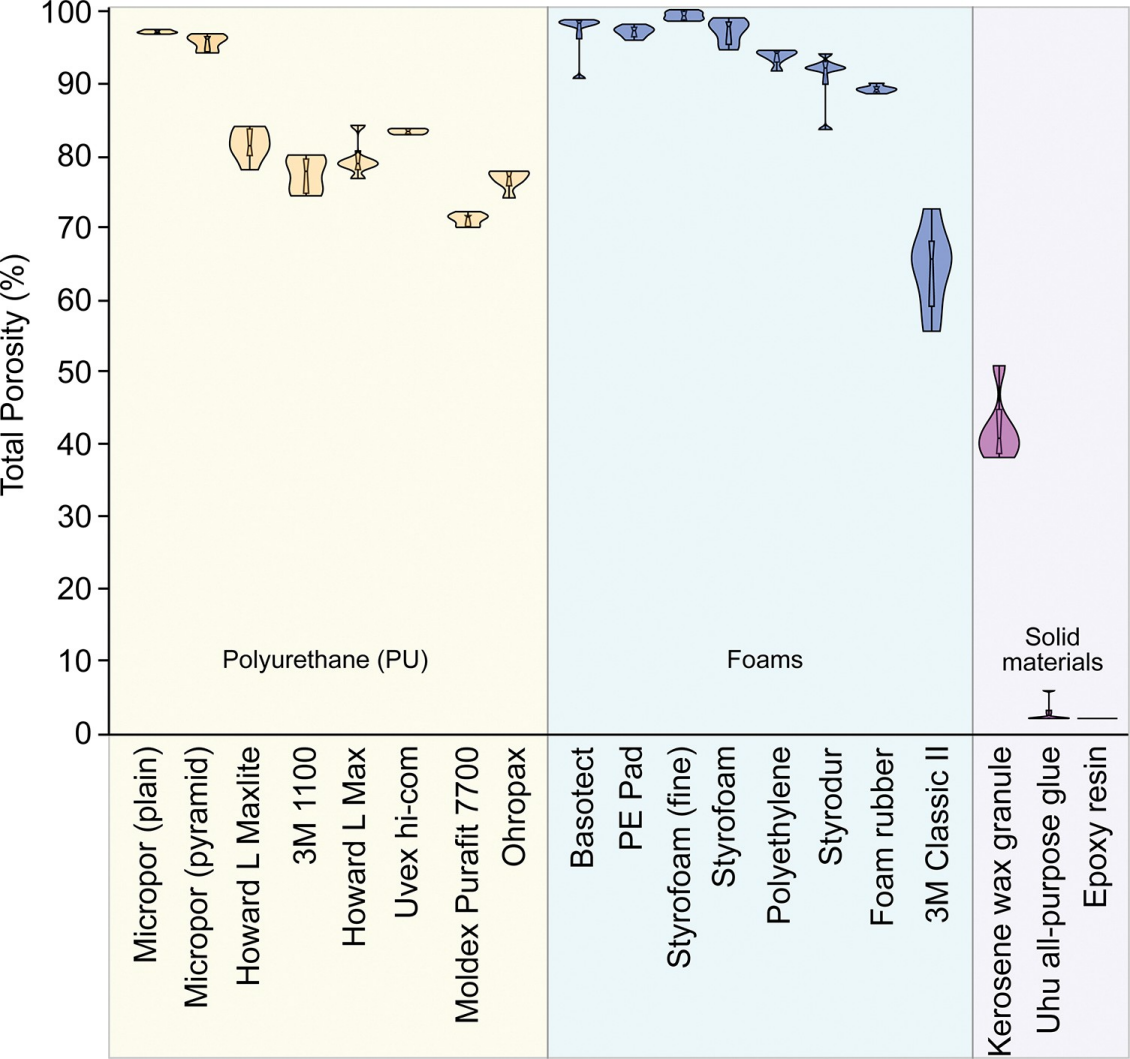
Illustration of the porosity of the tested fixation materials for micro-CT samples. Foams (blue) had on average the highest percentage of pores (open and closed pores) and solid materials (purple) had the lowest percentage of pores. Styrofoam (fine granular) is the most porous material tested, but it is more radiopaque than Basotect. (n = 6 per material).

PU samples are porous and under the constant analysis settings, on average between 17.1% to 35.9% of a digitally cut 8 mm^3^ cube (2x2x2 mm) was binarised (Fig 3) depending on the material, and these are thus clearly visible (Fig 4). With -632 HU (±20.20 HU) (Hounsfield Units), the Ohropax Soft (Ohropax GmbH, Wehrheim, Germany) were the most radiopaque of all PU samples examined (Fig 5). The most radiolucent PU were the Howard L Maxlite (Honeywell, Charlotte, USA) with -764 HU (±12.92 HU). A PU sample derived from a clinical earplug was barely visible in micro-CT images in preliminary tests with -961 HU (0.6% binarisation of the image) (S1 Fig). Micropor is another polyurethane foam with suitable properties, but with an X-ray density of -926 (±14 HU) HU and a porosity of 96.74% (±0.24%) it is more visible than Basotect foam or PE pads (shown in the chapter Foams). However, similar to the PE pad, Micropor has large air chambers (structure separation value 421 μm ±185 μm) with very few thick edge areas (Fig 4). A structure separation value is essentially the thickness of the spaces in the binarized image. The calculated average structure separation values for PU ranged from 55 μm (1100; 3M, St. Paul/Minnesota, USA) to 106 μm (Howard L Max). With an average porosity of 82.92% (±0.34%) Uvex hi-com have the highest proportion of pores and with 70.81% (±0.84%) Moldex Purafit 7700 (Moldex/Metric AG & Co. KG, Walddorfhäslach, Germany) have the lowest proportion of pores among the tested PU foam samples (Fig 6). The Howard L Maxlite, Howard L Max, and Ohropax earplugs consist of comparatively larger pores, but also show clearly visible walls of the air chambers in the binarised image (Fig 4). 3M 1100 and Uvex hi-com have significantly finer pores with thinner walls but are also clearly visible when binarised (Fig 4). Furthermore, some earplugs expanded for a few minutes after deformation, which easily leads to motion artefacts in a scan.

### Foams

Foams are the most radiolucent group of materials, with radiopacity between -943.96 HU (±6.76 HU) (Basotect®, BASF, Ludwigshafen, Hesse, Germany) and -470 HU (±45.84 HU) (3M Classic II) (Fig 5). Apart from the polyethylene (PE) pad, styrofoam and Micropor, the radiolucency of Basotect is significantly higher than that of all other samples (p: 0.019 to p: <0.0001) (Fig 5). In addition, foams have the highest porosity of the materials tested (Fig 6). The most porous material tested in this study is fine-grained polyfoam with a porosity of 98.98% (±0.62%). Styrofoam (fine-grained) was also the least visible material when the scan was binarised, with an average image fraction of 1% and -935 HU radiopacity. The PE pad consisting of air cushions is also among the most suitable fixation materials with -943.97 HU (±5.26 HU) and a porosity of 96.78% (±0.78%). In the binarised image, the edges of the air cushions are clearly visible (Fig 4), but due to their size, they are easy to process in a later image adjustment. In foams structure separation values ranged from 54 μm in the 3M Classic II (polymer foam) to 524 μm in the PE pad. The most radiopaque foams (Fig 5) in this study beneath the 3M Classic II are polyethylene (-894 HU ±9 HU), styrodur (-871 HU ±24 HU) and moss rubber (-874 HU ±5 HU). These materials also have the lowest porosity (88.9% to 93.4%) of the foams studied. However, these three materials are easy to form into specific shapes. Especially blocks made of soft polyethylene can be cut without forming dust or detaching crumbs. The lowest proportion of pores in foams was found in the 3M Classic II (porosity of 64.11% ±5.79%).

### Solid materials

Another strategy to fix specimens and subsequently binarise them is to use materials that are more radiodense than the probe instead of using radiolucent materials. However, some of these embeddings, such as all purpose glue or epoxy resin, are not reversible. All purpose glue is very radiopaque with 245.06 HU (±15.98 HU) (Fig 5). Together with a low porosity of 2.78% (±1.54%), this results in a high average binarisation of 97.2% (±1.54) (Fig 4). Structure separation is comparatively low at 20 μm (±22 μm). The properties of epoxy resin as a fixation material are comparable to all purpose glue. With -190.41 HU (±7.08 HU) it is one of the most radiopaque materials in this study (p: 0.0071 to <0.001) (Fig 5). The porosity is 2.10% (± <0.00%) and 97.9% (± <0.0%) of the image was binarised. A reversible alternative to general purpose adhesives and epoxy resin is the use of wax granules. These beads are removable in subsequent image processing and have an X-ray density of -639.69 HU (±30.60 HU). The porosity is 41.90% (±4.60%) and the structure separation is 178 μm (±49 μm). These values are strongly influenced by how the wax granules are compressed, as these mainly reflect the spaces between the granules. In the subsequent processing of the scan, an average of 58.1% (±4.6%) was binarised.

## Discussion

The main findings of our study were:

1. The different fixation strategies for samples in micro and nano CT result in a variety of imaging features.
2. For different types of probes there are specifically best suited fixation materials.
3. Filtering using thresholds when binarising the scan data allows both radiolucent and radiopaque fixation materials to be segmented.

The fixation materials proved having a wide variety of different features with the result that there is generally no best fixation material equally suited for all probes. It turned out, that different types of probes benefit from different fixation strategies, depending on their structure, density, durability, storage and water content. Since each sample has different fixation requirements, each micro-CT laboratory should have different materials in stock for optimal results.

Since all fixation materials in this study were tested under the same conditions, the scans took place dry in the reaction vessel surrounded by air. However, the following recommendations in descending order can also be applied to scans with samples in liquid. Please keep in mind that the materials shown here are uncompressed. Foams and PU can be significantly more radiopaque when compressed and produce more image artefacts.

### Styrofoam

Styrofoam is one of the most common materials used to support or fix samples in (micro-)CT due to its specific properties [5, 39–43]. This has also been shown in our measurements. In the analysis routine, styrofoam achieved 1% binarisation of the image (3.2% for the further large-grain styrofoam sample) and is thus excellently suited for the fixation of micro-CT samples. Styrofoam consists of a small amount of polystyrene (~2%) and mostly of air, which explains the X-ray density of -935 HU (as the X-ray density of air is defined as -1000 HU [44]). Furthermore, Styrofoam is a well-suited material for thermal insulation and thus suitable for cryo micro-CT measurements, for example [45]. A disadvantage of styrofoam can be its spherical composition when it has to be cut into shape and then disintegrates into beads. In addition, styrofoam is not suitable for all liquid media and can be easily dissolved.

### Basotect

Another highly recommended material for specimen fixation is Basotect foam, consisting of melamine resin. Due to the low absorptive properties for the X-ray, this material has already been used successfully several times in studies by Alba-Tercedor for simple erasure in segmentation [31, 46, 47]. Basotect is the most radiolucent material in this study (-944 HU) and thus comparable to the image quality of styrofoam. Due to the fine porosity, only a few remnants of the material are visible in the reconstructed image. When measuring more radiopaque samples with higher kV, such as bones, Basotect is usually no longer visible in the X-ray image. One advantage is that smaller shreds can also be detached for padding samples, although dust may form in the process.

### Polyethylen (PE)

Polyethylene was investigated in this study in the form of foam pads and air-cushion foil. We could not find any other studies in which polyethylene is used for embedding or fixing (micro-) CT specimens. As a foam pad, the material is slightly more radiopaque than Styrofoam or Basotect, but it can be cut into shape very well and expands again quickly when deformed, so that the probes are fixed without squeezing and no movement artefacts occur in the scan. Other easily cut materials in this study, such as foam rubber and Styrodur, are significantly more radiopaque than polyethylene with many clearly visible radiopaque particles (Figs 3 and 4). Polyethylene as an air cushion consists of very large pores compared to the other fixation materials and therefore large proportions of air only. As a result, the X-ray permeability is significantly higher compared to polyethylene foam pads. The partitions are easy to erase in the segmentation and the air chambers cushion the sample well. Therefore, we recommend fixation with polyethylene especially for sensitive samples.

### Micropor (polyurethane)

The polymer Micropor contains polyurethane (polyol and isocyanate foamed with carbon acid). Similar to the PE pad, Micropor consists of large air cushions, but their walls are more visible (more radiopaque). As a result, the binarised image portion of the fixation material is slightly higher than Basotect, PE pads, or Styrofoam (Fig 4). Micropor is still very well suited for the fixation of micro-CT specimens. If this material is used for fixation, it is still clearly visible in the reconstructed image, depending on the voltage applied. Therefore, this material usually has to be removed in the image processing by segmentation instead of a simple threshold binarisation. However, since the partitions of the air cushions are very homogeneous, this should not pose any major difficulties.

### Glue and resin

An alternative to using radiolucent materials is to fix the sample in a medium that is as homogeneous as possible or quite radiopaque. Thus, segmentation over a threshold value, like with foams, is usually possible. The only difference is that radiopaque materials are masked out. However, it can also reduce the contrast of the image if the sample is surrounded by too much radiopaque material. Epoxy resin or all-purpose glue with a very homogeneous image (Figs 3 and 4) have proven to be suitable as embedding material. This fixation is usually not reversible and must therefore be used with care. Besides epoxy resin and paraffin wax, which was also investigated in this study, Strotton et al. [48] were able to scan samples fixed in agar. However, the scan was compromised by rising air bubbles. Reversible alternatives are modelling wax, Surgident and PluLine, or Leit-C-Plast (S2 Fig). These materials can contain very radiopaque inclusions (S3 Fig) and easily form air pockets during fixation and are therefore rather poorly suited for micro-CT.

### Kerosene wax granules

Lightweight granules made of kerosene wax have the advantage that they can be easily tilted around a very delicate sample. In this way, the sample can be securely mounted in a reaction vessel, for example, without having to squeeze it. By heating, the wax granules can also be melted and glued together. A disadvantage of this method is that wax is considerably more radiopaque than foams and must be segmented in the image processing.

### Polyurethane (derived from earplugs)

In preliminary tests, clinical polyurethane (S1 and S2 Figs) proved to be a particularly good fixation material for micro-CT specimens. Unfortunately, these results could not be achieved with any other earplug tested, which mostly consists of polyurethane except the 3M Classic II in this study. The other polyurethane samples are nevertheless well suited for fixing reaction vessels in plastic tubes. However, they must have time to expand, otherwise, movement artefacts will occur during the scan. Compared to the foams tested, such as Basotect and styrofoam, polyurethane from earplugs is less suitable as a fixation material. They are more radiopaque than foams (Fig 5), which is probably due to their lower porosity (Fig 6). As a result, the binarised image portions are also high (Figs 3 and 4) and difficult to segment. At least earplugs are available nearly everywhere and inexpensive.

## Conclusion

After all, there are several ways of fixation for each sample and often no perfect solution. For instance, Swart et al [49] were able to fix and evaluate several flies on top of each other with cotton wool, even though this material is somewhat more X-ray dense than foam in our results (S2 Fig) and can be prone to artefacts depending on the manufacturer (S1 Fig). Likewise, samples (often insects) are simply attached to cardboard with pins or plasticine [20]. Provided that this does not limit the area of interest, these are also suitable ways of fixing specimens.

In addition to fixation materials commonly referred to in the literature, such as styrofoam and Basotect foam, other materials like polyethylene air cushions or Micropor foam are also ideally suited for fixing micro-CT samples. Alternatively, PU from ear plugs or similar sources can be used, but these are usually more radiopaque and will continue to expand for several minutes. To facilitate segmentation, the possibility of contrasting the probe [19, 33, 34, 49, 50] should also be considered.

## Supporting information

S1 Fig Rendered images of further porous samples. Grey values from the dynamic range of 0 to 0.03 are coded as colour values (0 to 255). The binarised image portion of the sample is shown as a percentage for each material (n = 1 per material). Low binarised values are less visible in the evaluation than high values. Scale: 1 mm.
(TIF)

S2 Fig X-ray density in Hounsfield units of further fixation materials with only one probe. An earplug from an unknown manufacturer was the most radiolucent material in the entire study. It had almost the same appearance as the 3M 1100, but unfortunately could not be identified. Samples below the dotted line could also be good fixation materials for μCT samples. (n = 1 per material).
(TIF)

S3 Fig Rendered images of further solid samples. Grey values from the dynamic range of 0 to 0.03 are coded as colour values (0 to 255). The binarised image portion of the sample is shown as a percentage for each material (n *=* 1 per material). Low binarised values are less visible in the evaluation than high values. Scale: 1 mm.
(TIF)
