# Supplementary figures and images for: Analysis of fixation materials in micro-CT: It doesn’t always have to be styrofoam
Source: PLoS One. 2023 Jun 14;18(6):e0286039. doi: 10.1371/journal.pone.0286039 (PMC10266650; doi:10.1371/journal.pone.0286039)

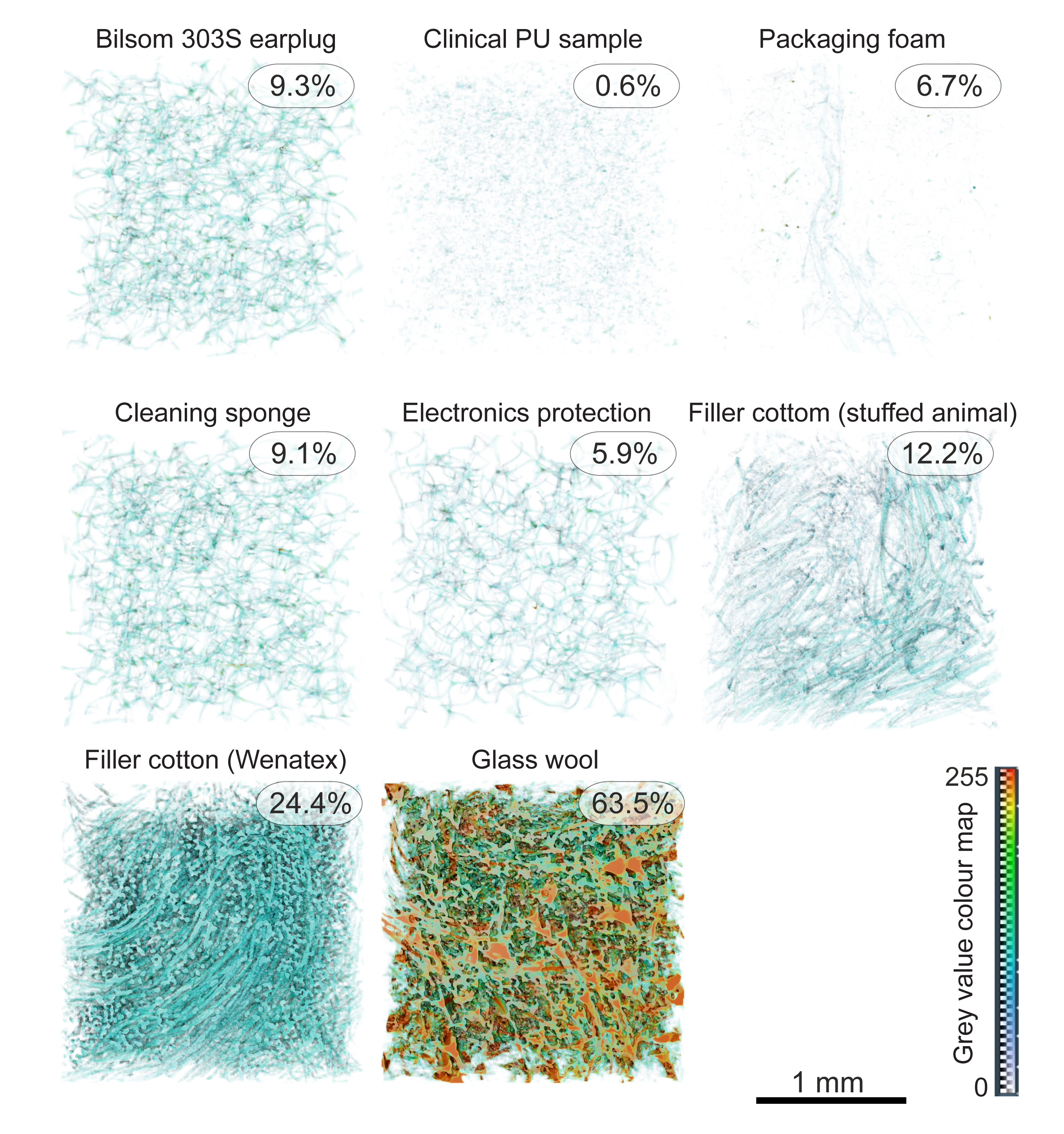

Supplement: S1 Fig — Grey values from the dynamic range of 0 to 0.03 are coded as colour values (0 to 255). The binarised image portion of the sample is shown as a percentage for each material (n = 1 per material). Low binarised values are less visible in the evaluation than high values. Scale: 1 mm. (TIF) [file pone.0286039.s001.tif]

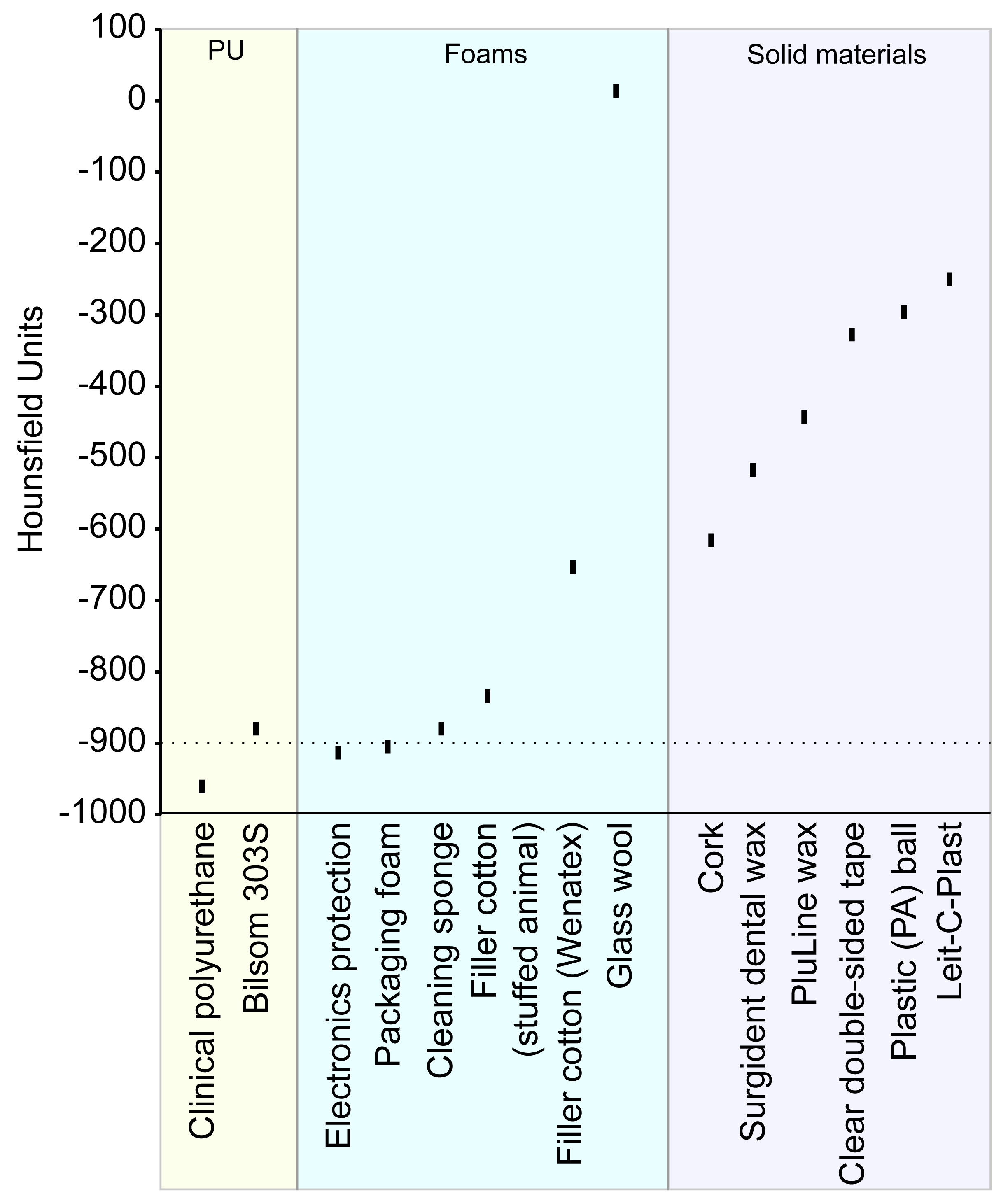

Supplement: S2 Fig — An earplug from an unknown manufacturer was the most radiolucent material in the entire study. It had almost the same appearance as the 3M 1100, but unfortunately could not be identified. Samples below the dotted line could also be good fixation materials for μCT samples. (n = 1 per material). (TIF) [file pone.0286039.s002.tif]

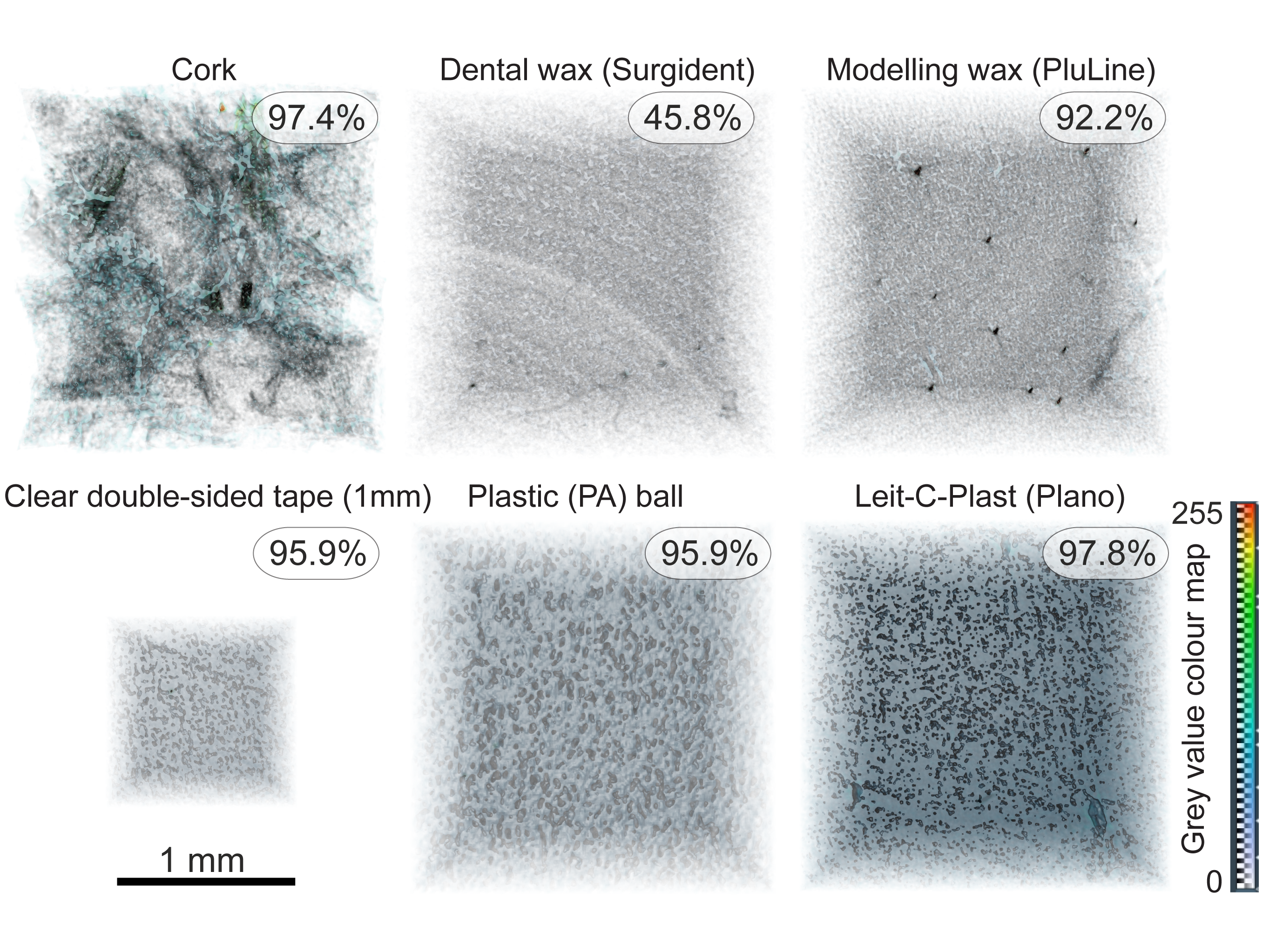

Supplement: S3 Fig — Grey values from the dynamic range of 0 to 0.03 are coded as colour values (0 to 255). The binarised image portion of the sample is shown as a percentage for each material (n = 1 per material). Low binarised values are less visible in the evaluation than high values. Scale: 1 mm. (TIF) [file pone.0286039.s003.tif]
